# The Role of Liquid–Liquid Phase Separation in Actin Polymerization

**DOI:** 10.3390/ijms24043281

**Published:** 2023-02-07

**Authors:** Olga I. Povarova, Iuliia A. Antifeeva, Alexander V. Fonin, Konstantin K. Turoverov, Irina M. Kuznetsova

**Affiliations:** Laboratory of Structural Dynamics, Stability and Folding of Proteins, Institute of Cytology, Russian Academy of Sciences, 4 Tikhoretsky Ave., 194064 St. Petersburg, Russia

**Keywords:** actin, actin polymerization, actin-binding proteins, liquid–liquid phase separation (LLPS), coacervate, membrane, signaling proteins

## Abstract

To date, it has been shown that the phenomenon of liquid–liquid phase separation (LLPS) underlies many seemingly completely different cellular processes. This provided a new idea of the spatiotemporal organization of the cell. The new paradigm makes it possible to provide answers to many long-standing, but still unresolved questions facing the researcher. In particular, spatiotemporal regulation of the assembly/disassembly of the cytoskeleton, including the formation of actin filaments, becomes clearer. To date, it has been shown that coacervates of actin-binding proteins that arise during the phase separation of the liquid–liquid type can integrate G-actin and thereby increase its concentration to initiate polymerization. It has also been shown that the activity intensification of actin-binding proteins that control actin polymerization, such as N-WASP and Arp2/3, can be caused by their integration into liquid droplet coacervates formed by signaling proteins on the inner side of the cell membrane.

## 1. Introduction

Actin exists in nature in a monomeric globular form (G-actin) and a polymeric fibrillar form (F-actin). In the cytoplasm of cells, F-actin, which is the main part of the cytoskeleton, has a functional role. The actin cytoskeleton is necessary for a variety of processes in cells, including the establishment of cell polarity, the activation of cell migration, the launch of cytokinesis, and the positioning of intracellular organelles. Despite extensive research during the last 100 years, there is no complete understanding of the dynamic nature of the cytoskeleton and its regulation in cells and tissues. Recent work has shown a significant role of biomolecular condensates in the regulation of the formation, functioning, and assembly/disassembly of the cytoskeleton [1,2,3,4,5].

The presence of coacervates in protoplasm was also observed a long time ago [6]; however, the significance and universality of this phenomenon of the most diverse intracellular processes became clear only after the work of Brangwynne et al. [7]. The study of LLPS, and its role in cell life, is one of the most rapidly developing areas of molecular and cellular biology. It becomes obvious that the phase separation of biomacromolecules plays a significant role in almost all intracellular processes.

It is believed that intracellular liquid–liquid phase separation leads to the formation of coacervates (also called droplets, membraneless organelles, bodies, and granules), which do not have a membrane separating them from the rest of the cellular space, and which include proteins and nucleic acids. A key role in the formation of membraneless organelles is played by proteins containing intrinsically disordered regions (IDRs), which are mainly scaffold proteins of these structures [8,9].

The IDRs of proteins are important for the fluid-like behavior of coacervates. Studies have shown that proteins rich in IDRs are more likely to separate into phases that retain the dynamic properties of the IDPs forming them [10]. Post-translational modifications (PTMs), such as phosphorylation and SUMOylation, also have a significant impact on LLPS, allowing cells to dynamically regulate LLPS in response to various cellular signals [11,12,13].

A high degree of multivalence, also inherent in IDPs, is necessary for recruiting other components into membraneless organelles and thus increasing their local concentration [14]. Liquid–liquid phase separation (LLPS) has been recognized as a general cellular mechanism of control for the spatiotemporal dynamics of many important signaling pathways [14,15].

The most important, functionally significant features of membraneless organelles are that only weak interactions are necessary for their formation, they are easily assembled and disassembled after performing their function, the absence of a rigid membrane allows them to freely exchange molecules with the environment, and weak impacts can significantly change their properties. At the same time, mature, membraneless organelles can be complex structures that include hundreds of protein molecules, including globular proteins and RNA [16]. The components of condensates are subdivided into scaffold proteins responsible for phase separation and client proteins, which are included in membraneless organelles to fulfill their function. In coacervates, the concentration of client proteins, including actin, can significantly exceed the concentration in the environment surrounding the organelle.

It is well known that a high concentration of actin is required in a solution to initiate the polymerization of actin monomers. This is primarily due to the fact that the formation of a polymerization nucleus requires the interaction of at least three actin monomers [17]. Coacervates working as biological reactors can contribute to an increase in the likelihood of this event.

## 2. Model Coacervates as Reactors of Actin Polymerization Initiation

The possibility of actin polymerization through increasing its concentration in model coacervates was shown for the first time in prior work [18], which aimed to demonstrate the possibility of creating a bioreactor that provides a significant increase in the reaction rate. It is known that charged homopolymers (polyelectrolytes) form liquid phases through complex coacervation [19] and can include charged proteins [20,21,22] and small molecules. To demonstrate the operation of a bioreactor based on poly-L-lysine (pLK) polycation and poly-(L, D)-glutamic acid (pRE) polyanion, two proteins were used: monomeric G-actin and bovine serum albumin (BSA) [18]. G-actin and BSA monomers are globular proteins; they are similar in size (42 and 66 kDa, respectively) and carry a comparable negative charge (isoelectric points of 5.23 and 5.60, respectively).

**Figure 1:**
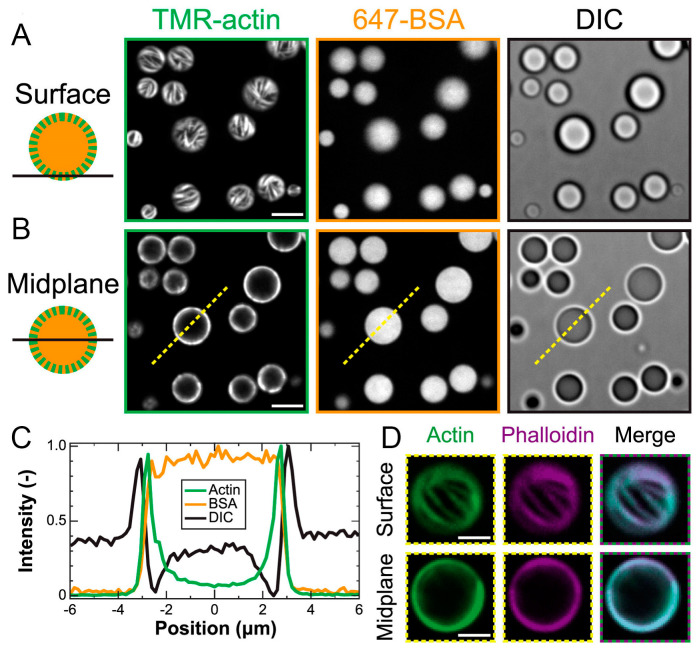
Actin polymerization in the coacervates formed by poly-L-lysine (pLK) and the polyanion poly-(L,D)-glutamic acid (pRE) at the phase boundary. (**A**,**B**) Microphotographs, obtained using confocal fluorescence microscopy (left and middle) and differential interference contrast (DIC) microscopy (right), show polypeptide coacervates containing TMR-actin (green) and Alexa 647-BSA (orange). The focal plane is at the interface of the coacervates and the substrate (surface (**A**)) or near the midplane of the droplet (**B**), indicated by the dashed yellow line. Scale bar, 5 mm. (**C**) Normalized intensity line scans are provided along the dashed yellow lines, as indicated in (**B**). (**D**) False-colored fluorescence images are provided in (**A**,**B**) from the surface (upper row) and midplane (bottom row). The figure represents panels (A), (B), and (D) of Figure 1 and panels (C) and (D) of Figure 2 from the article McCall et al., Biophysical Journal 114, 1636–1645, © 2018 [18].

The proteins were visualized using fluorescently labeled protein-TMR-actin (green) and Alexa-647-BSA (orange). It turned out that both proteins accumulated in the created coacervates model; moreover, actin formed fibrils that were localized at the phase boundary, and BSA filled the coacervates evenly (Figure 1). Using fluorescently labeled phalloidin (Alexa Fluor 647-phalloidin)—a small, uncharged toxin known to bind specifically to F-actin—fibrils, formed by actin, near the coacervate surface were shown to be F-actin (Figure 1D). The extent to which the coacervate microenvironment influenced the rate of actin polymerization was investigated by recording pyrene fluorophore fluorescence.

A study of the polymerization of 1.5 mM of actin in a solution showed the presence of a characteristic lag phase, indicating a slow stage of filament nucleation [23] followed by a phase of rapid growth and then saturation after reaching a steady state [24]. At an actin concentration of 1.5 mM, the initial lag phase is usually 10 min, and the steady state is reached after 120 min. The presence of pLK/pRE coacervates eliminates the lag phase, and a steady state is established within 10 min. Thus, actin incorporation in pLK/pRE coacervates significantly stimulated its polymerization.

The authors showed that the mechanism underlying the increased assembly rate is an increase in the local concentration of actin in coacervates. The threshold monomer concentration/critical concentration required for the polymerization of Mg-ATP-actin is 0.1 mM [25]. If actin is concentrated 30-fold in coacervate droplets, then the expected assembly of actin within coacervates should be observed at total actin concentrations of 0.003 mM. However, F-actin formation in coacervates was experimentally observed at total actin concentrations of 0.05 mM. Interestingly, a peripheral shift of F-actin in pLK/pRE coacervates was observed at all actin concentrations studied—even at the lowest concentration (0.01 mM), at which the filaments are almost indistinguishable.

A little later, actin polymerization was examined in a model of the two-phase poly(ethylene glycol) (PEG)/dextran system [26]. It was shown that globular actin was distributed evenly between the phases, while F-actin was concentrated inside the droplets and F-actin bundles were distributed along the periphery of the droplets, deforming them. In addition, it was shown in this work that, in the two-phase PEG/dextran system, the threshold concentration of KCl (which causes actin polymerization) decreased by about one order of magnitude, and the threshold concentration of MgCl_2_ (which causes the formation of F-actin bundles) decreased by a third [26].

The authors of [18] consider three physical mechanisms of F-actin localization on the periphery of coacervates that do not exclude each other: the bulging of filaments, the depletion of macromolecules, and interfacial adsorption. However, it is noted that the results available to date do not provide a definitive answer for what determines the peripheral localization of F-actin; this could be an interesting problem for future research.

## 3. Initiation of Actin Polymerization from abLIM1 Coacervates

**Figure 2:**
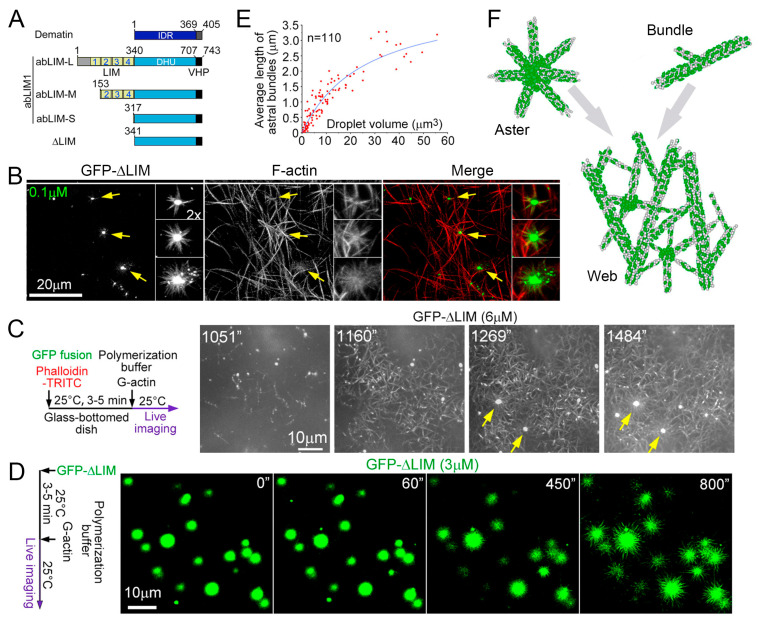
ΔLIM induces asters and webs of F-actin bundles in vitro. (**A**) Isoforms of human abLIM1. (**B**) Effects of GFP-ΔLIM on F-actin organizations in vitro. Arrows indicate representative astral structures. This presents the results of experiments in which the GFP-ΔLIM concentration was 0.1 μM. (**C**) The scheme of the experiment and the results of live imaging. The final concentration of G-actin was 6 μM. Phalloidin-TRITC (final concentration: 4 μM) was used to label F-actin. Representative time-lapse images showing the formation of His-GFP-ΔLIM-induced actin webs. Z-stack images, at 1 μm intervals, were captured for the GFP autofluorescence to cover a depth of 6 μm close to the bottom of the substratum via spinning disk microscopy at ~3.6 s intervals. The time started immediately after the addition of G-actin. Arrows denote two asters integrated into the web. (**D**) Massive actin polymerization from liquid droplets of GFP-ΔLIM. The experiments were performed without phalloidin-TRITC to show that the aster formation was not due to the presence of phalloidin. (**E**) Positive size–length correlations between the GFP-ΔLIM droplets and astral F-actin bundles. The size (volumes) of each droplet and the mean length of its astral bundles were measured from time-lapse images. (**F**) Illustrations showing ΔLIM (green)-induced F-actin-based asters, bundles, webs, and their relationships. The figure represents panels A of Figure 1; line 5 of panel B; panels E, F, and H of Figure 2; and panels A and C of Figure 4 from Yang et al., PNAS 119 (29), e2122420119 [27]. Copyright © 2022 the Yang et al., published by PNAS. This article is distributed under Creative Commons Attribution-NonCommercial-NoDerivatives License 4.0 (CC BY-NC-ND).

For the first time, the phase separation of the actin-binding protein, abLIM1, which leads to the formation of coacervates (i.e., reactors for the polymerization of cortical actin) was shown in work by Yang et al. [27]. The nonerythroid actin-binding protein, abLIM1, is known to be critical for stable interactions between the plasma membrane and the actin cortex under mechanical stress. This actin-binding protein has a C-terminal region that is 40% identical to dematin, an erythroid protein [28,29], which consists of a large, N-terminal, intrinsically disordered region (IDR) and a short actin-binding region. This region is identical to the villin headpiece (VHP) domain [30,31,32,33]. It is known that abLIM1 is expressed as three isoforms; namely, the long (abLIM-L, which is very rare), medium (abLIM-M, the widely expressed main isoform), and short (abLIM-S, a dematin-like isoform) isoforms [29]. These isoforms differ in a number of N-terminal LIM domains that mainly serve in protein–protein interactions [34]. The proteins abLIM1 and dematin are important for the stable attachment of the cortex to the plasma membrane in non-erythroid cells [28] and erythrocytes [31,35,36], respectively (Figure 2A).

Previously, it was shown that bacterially expressed His-tagged GFP-ΔLIM (3 μM) induces the formation of dense networks of bound F-actin in vitro [28], such as dematin [30], and asters ~10 μm in diameter. Usually, asters contain an amorphous core involving His-GFP-ΔLIM, from which numerous actin filaments radiate, decorated with GFP-ΔLIM. It was shown that the asters’ sizes depended upon ΔLIM concentration, and aster fibers contained densely packed actin filaments (Figure 2C). This allowed the authors of the work [27] to suggest that ΔLIM condensates could serve as the centers of the actin filament asters’ organization in vitro. To prove this idea, it was necessary to rule out the presence of F-actin prior to aster formation (Figure 2C).

To this end, G-actin in a storage buffer (containing 0.2 mM of CaCl2) was mixed with GFP-ΔLIM (6 μM) followed by an actin polymerization buffer. Then, TRITC phalloidin (tetramethylrhodamine B isothiocyanate) was used to visualize F-actin. It appeared that GFP-ΔLIM-decorated actin bundles, especially GFP-ΔLIM punctate condensates, were shown to gradually emerge after about 15 min and expand rapidly with time (Figure 2C).

The network (web) of actin fibrils turned out to be quite stable and remained preserved for hours without signs of disassembly. The web had a thickness of 6 to 7 μm and was located above an array of His-GFP-ΔLIM condensates at the bottom of the substrate, while numerous F-actin asters of various sizes floated in the solution along with sporadic bundles. Thus, it was shown that ΔLIM was capable of the self-organization of asters and webs of actin bundles in vitro [27] (Figure 2F).

A comprehensive study of the properties of ΔLIM condensates in astral centers has been made (Figure 2B), and it has been proven that they have the properties of coacervates. To this end, in particular, the change in the turbidity of the solution with a change in temperature was monitored [27]. Increasing the temperature of the GFP-ΔLIM samples from 0 °C to 25 °C over 5 min resulted in an increase in turbidity. Moreover, the turbidity was reversible [27]. In addition, droplets were shown to increase in both size and density depending on the concentration of GFP-ΔLIM and could be clearly observed at a concentration of 1 μM. They could easily merge into larger ones, which also confirms their liquid properties. Fluorescence recovery after photobleaching (FRAP) assays revealed a rapid recovery of droplet fluorescence, indicating a dynamic exchange of ΔLIM molecules with the environment. Finally, in the presence of polyethylene glycol (PEG), a crowding agent known to promote the separation of protein phases [37,38], droplet formation was observed even at 8 nM [27]. The ability to achieve the phase separation of various modifications of His-GFP-DHU-VHP, including His-GFP-DHU and His-GFPHis-GFP-VHP, was considered. The results of these studies concluded that the IDR region homologous to dematin mediated LLPS.

**Figure 3:**
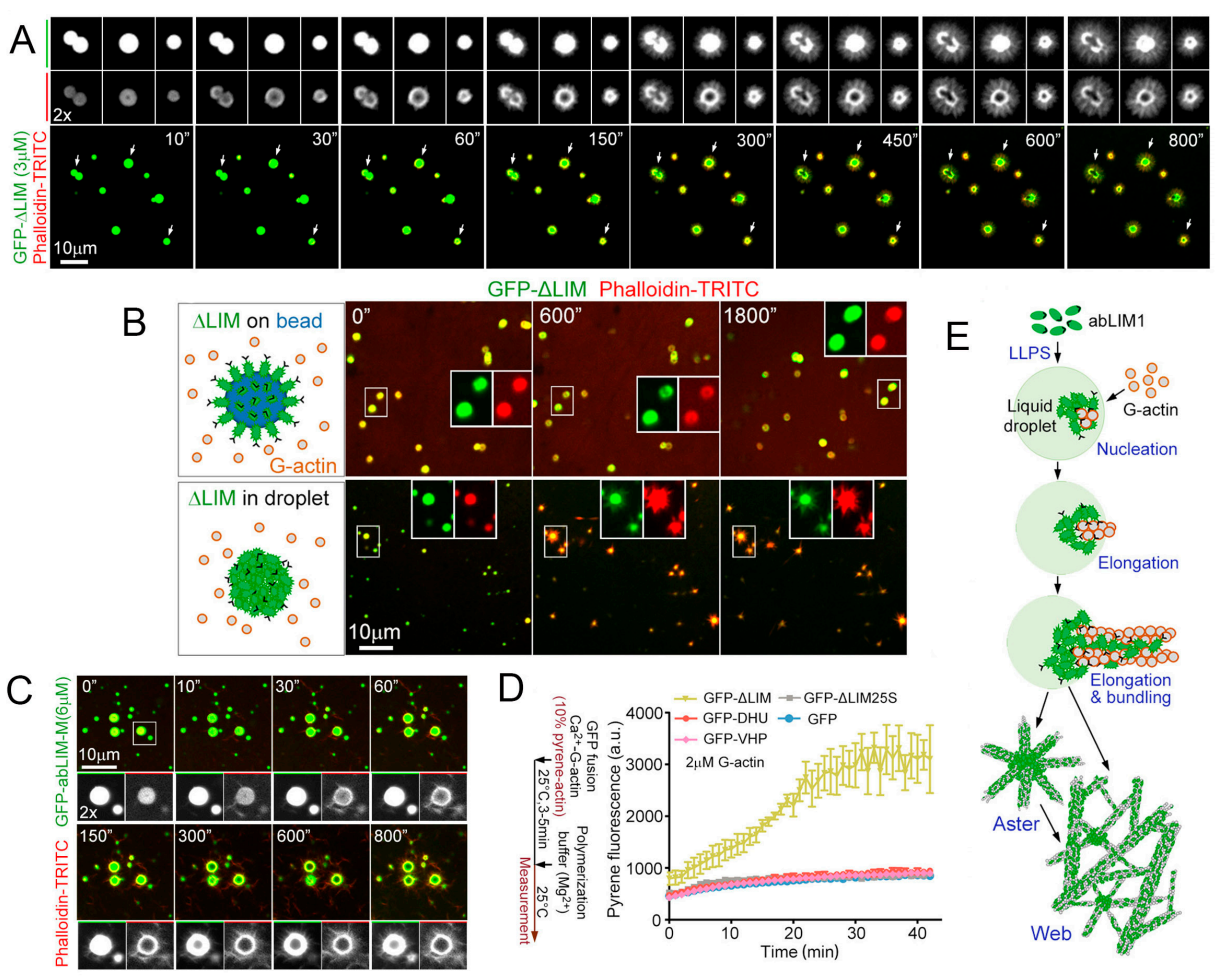
Nucleation, elongation of polymerization, formation of asters, and F-actin filaments in the presence of ΔLIM or abLIM-M liquid droplets. (**A**) Intense actin polymerization from GFP-ΔLIM liquid droplets in real time. The initial moment of time was taken as the time when G-actin was introduced into the actin polymerization buffer containing preliminarily formed GFP-ΔLIM liquid drops. (**B**) F-actin does not polymerize from GFP-ΔLIM immobilized with an anti-GFP antibody on magnetic beads (upper panel); however, in the presence of preformed His-GFP-ΔLIM droplets, actin filaments grow rapidly (lower panel). (**C**) abLIM-M liquid droplets can also generate asters. Although the LIM domain inhibits droplet formation, GFP-abLIM-M forms droplets in the presence of 1% PEG. Drops of GFP-abLIM-M, as well as GFP-ΔLIM, induce aster formation after the addition of G-actin. (**D**) G-actin polymerization requires the presence of GFP-ΔLIM or GFP-abLIM-M droplets. Individually, neither GFP-DHU (IDR domain) nor GFP-VHP (actin-binding domain) affects the rate of actin polymerization. (**E**) A model illustrating the role of abLIM1 in actin polymerization. At least, its abLIM-S or abLIM-M isoform undergoes LLPS to form condensates that promote actin concentration, initiate polymerization, and cross-link actin filaments to form asters and sporadic bundles that further develop into the F-actin web. The figure represents panels B, E, and G of Figure 4 and panels C and F of Figure 5 of Yang et al., PNAS 119 (29), e2122420119, 2022, [27]. Copyright © 2022 Yang et al., published by PNAS. This article is distributed under Creative Commons Attribution-NonCommercial-NoDerivatives License 4.0 (CC BY-NC-ND).

Finally, since aromatic amino acids (Fs and Ys) are known to be critical for the LLPS of some IDRs [14,27,39], DHU mutants that had a serine (S) residue instead of aromatic residues were investigated, and it was shown that that the resulting mutants, DHU25S and ΔLIM25S, did not form coacervates. Thus, it was proven that ΔLIM can, indeed, be separated into liquid droplets due to its IDR. In order to prove that ΔLIM liquid droplets can function as actin polymerization sites for the direct production of de novo radial actin filaments, GFP-ΔLIM liquid droplets were examined through live imaging immediately after the addition of G-actin. Numerous hair-like protrusions were shown to continuously grow from droplets (Figure 2D,E), indicating the massive nucleation and elongation of F-actin. To prove that the rays were formed by F-actin, the slides were stained with 4 μM of phalloidin-TRITC. Although the presence of phalloidin can potentially stabilize actin filaments, it does not affect the rate of actin polymerization [40] and, thus, does not interfere with the process of aster formation. Indeed, phalloidin-TRITC rapidly congressed to the outer surface of the droplets within 30 s after the G-actin addition, after which, GFP-positive F-actin arrays gradually appeared around all of the droplets (Figure 2A,D). It is noteworthy that some of the drops gradually became hollow during the growth of the aster, which suggests that the elongation of the astral bundle absorbs the contents of the drops. Accordingly, larger droplets produced longer bundles of astral actin (Figure 2D and Figure 3A).

It was also shown that aster formation was not observed in the presence of Cytochalasin D (CytoD) or LatrunculinA (LatA), drugs that inhibit actin polymerization and prevent actin assembly in the plus-end [24] or sequestering G-actin [41].

To find out whether the formation of aster was simply the result of a directed arrangement of abLIM1 molecules on a spherical surface and not their phase separation, a control experiment was carried out with magnetic beads. To perform this experiment, HEK293T cells were overexpressed with GFP-ΔLIM and concentrated with anti-GFP, antibody-conjugated magnetic beads ~1.5 µm in diameter. Thus, the ΔLIM molecules on the beads were oriented so that their actin-binding VHP domain was directed radially from the center of the bead. Direct imaging showed that the fluorescence intensity of the GFP beads was comparable to that of similarly sized liquid GFP-ΔLIM droplets (Figure 3B), suggesting a similar density of GFP-ΔLIM molecules on the surface of the beads and the droplets. However, in the presence of G-actin, the beads could not produce visible actin filaments, while liquid droplets induced aster formation. Therefore, the formation of ΔLIM coacervates is necessary for aster formation [27].

Thus, the authors of the work [27] proved that ΔLIM, consisting mainly of a disordered fragment of DHU, can form coacervates, which are actin polymerization reactors, due to LLPS. At the same time, it was shown that both DHU and VHP are necessary for actin aster formation since no aster formation was observed in experiments with GFP-DHU liquid droplets.

Since actin-binding proteins are the entire abLIM family, it was necessary to find out how the ordered LIM fragment affects LLPS and whether these proteins could form coacervates. The tendency to precipitate His-GFP-abLIM-L [28] made it difficult to perform experiments with this form. However, His-GFP-abLIM-M, a widely expressed major isoform (Figure 1A) [29], was soluble, so the study of whether its liquid droplets could also generate aster was carried out with this isoform. It was found that GFP-abLIM-M did not phase separate, even at 105 μM, consistent with the inhibitory role of the LIM region. However, it was subjected to LLPS at 6 μM in the presence of 1% PEG (Figure 3C).

Live imaging showed that, after the addition of G-actin, liquid droplets mediated aster formation in a similar manner (Figure 3C). Therefore, the separated abLIM-M and abLIM-S phases were able to facilitate local actin polymerization and binding to form complex aster.

In [27], the rate of actin polymerization in the presence of abLIM1 was also estimated. For this, the authors used the fluorescence of pyrene. An essential increase in the actin polymerization rate was shown in the presence of abLIM1. It is known that the stage limiting rate of actin polymerization is the formation of nuclei, i.e., the formation of initial actin trimers [42,43]. To quantify how abLIM1 promotes actin nucleation, the G-actin concentration was chosen to be 2 μM, which excludes spontaneous actin nucleation [44,45]. This made it possible to evaluate the activity of nucleation mediated by nucleators [46,47]. It turned out that while pyrene fluorescence in GFP samples only slightly increased over time; it steadily increased in 3 μM GFP-ΔLIM samples and stabilized after about 25 min (Figure 3D). The steady-state pyrene intensity was more than five times higher than that of the GFP samples (Figure 3D). However, separately, neither GFP-DHU nor GFP-VHP had a clear effect on actin polymerization kinetics compared to GFP (Figure 3D), confirming the need for both regions. As expected, the GFPΔLIM25S that did not undergo LLPS was unable to stimulate actin polymerization (Figure 3D).

Thus, based on all in vitro studies, it can be concluded that LLPS-induced ΔLIM condensates are capable of strongly stimulating actin nucleation. The resulting actin filaments are then stitched together to form stable asters and sporadic beams that further develop into stable networks (Figure 3E).

Condensates of abLIM1 likely move along actin filaments to regionally nucleate and cross-link actin filaments together to self-organize elastic, interconnected networks of F-actin cortical bundles so that cells properly resist mechanical stress. The tendency of F-actin polymerizing arrays to consume liquid droplets and the positive correlation between the length of actin astral bundles and the size of liquid droplets suggest fluid fluidity of ΔLIM and abLIM-M along F-actin in vitro. In [27], it was concluded that the absence of the F-actin association of the phase-defective ΔLIM25S and abLIM25S-M mutants, the appearance of liquid droplets of abLIM-M and ΔLIM during actin depolymerization in cells, their resorption into repolymerizing actin cytoskeletons, and their constant propagation along intracellular F-actin bundles confirmed that the intracellular molecules, abLIM-M and abLIM-S, associated with F-actin were also form coacervates. Moreover, previous studies demonstrate that dense, interconnected cortical actin networks in RPE1 and U2OS cells become sparse and rich in thick linear filaments when abLIM1 is depleted, leading to membrane swelling during cell proliferation or migration [28]. In addition, because abLIM1 is highly enriched in the Z-disc of sarcomeres [29,48], it may also assist in the construction of actin networks in striated muscle cells through the nucleation and cross-linking of actin filaments in the Z-disc. This is also consistent with the ΔLIM-induced efficient polymerization of Ca^2+^-G-actin in the absence of Mg^2+^.

## 4. LLPS of Signaling Proteins Leads to the Formation of Coacervates Concentrating Actin-Binding Proteins, N-WASP, Arp2/3, and Cortactin

A recent review on the role of phase transitions in the formation of the cytoskeleton [3] analyzed the role of phase transitions in actin polymerization and briefly mentioned several studies showing that the phase separation of signaling proteins modulates the functioning of proteins associated with actin polymerization.

It was shown in [1] that the phase transition of the neuronal Wiskott–Aldrich syndrome protein (N-WASP)—which interacts with its biological partners, Nck and phosphorylated nephrin—leads to the formation of coacervates, which include complexes (actin-related proteins), Arp2/3, which are actin nucleation factors. It has been shown that actin polymerization increases dramatically when coacervates of signaling proteins are formed and decreases when the LLPS process or the interaction of Arp2/3 with N-WASP is disrupted. In [49], a mechanism was proposed for the intensification of the Arp2/3 complex in coacervates. It has been shown that the phase separation of Nephrin/Nck/N-WASP signaling proteins on lipid bilayers leads to an increase in the residence time of N-WASP and the Arp2/3 complex on the membrane and, therefore, intensifies actin assembly.

Another example of condensates that enhance actin polymerization is activated T-cell receptors [2]. The authors biochemically recreated a 12-component signaling pathway on model membranes, starting with T-cell receptor (TCR) activation and ending with actin assembly. When TCR phosphorylation was triggered, signaling proteins spontaneously separated into fluid-like clusters that facilitated signaling both in vitro and in human Jurkat T cells. The reconstituted clusters were enriched in kinases, but excluded phosphatases and enhanced the assembly of actin filaments by recruiting and organizing actin polymerization regulators, N-WASP-Arp2/3. Thus, it was demonstrated that the LLPS of proteins can create separate physical and biochemical compartments that facilitate signaling.

Another example of this phenomenon was considered in [50], where signaling in synapses was considered. Obviously, the correct formation and rapidity of responses to synaptic stimulation are fundamental for the functions of the mammalian brain; however, the molecular basis that governs the formation and modulation of separated synaptic ensembles has, so far, remained unclear. Using a biochemical reconstruction approach, the authors showed that post-synaptic scaffold proteins at physiological concentrations can form highly condensed, self-assembling postsynaptic density protein (PSD)-like assemblies via liquid–liquid phase separation (LLPS). Such PSD scaffold condensates can cluster glutamate receptors, incorporate synaptic enzymes, and promote the formation of actin bundles; however, they do not allow gephyrin, which is the main scaffold protein for almost all inhibitory synapses, to pass through.

When creating model coacervates, the authors used the main PSD scaffold proteins, including PSD-95, GKAP, Shank, and Homer, which serve to connect ion channels/receptors on the postsynaptic plasma membrane to the actin cytoskeleton in the PSD cytoplasm. It is known that Shank directly interacts with regulatory proteins of the actin cytoskeleton, such as cortactin [51] and subunits of the Arp2/3 complex [52].

Cortactin has an N-terminal acidic domain that binds to and stimulates the Arp2/3 complex, followed by tandem cortactin repeats (CR) that bind to F-actin, and a C-terminal SH3 domain that binds to Shank3. It was shown that when cortactin, the Arp2/3 complex, and G-actin were introduced into the 63 PSD system assembled on lipid bilayers, polymerized structures of F-actin began to appear in the condensed PSD assemblies 15 min after the onset of the phase transition. Actin bundles colocalized with PSD condensates gradually became thicker and longer. Phalloidin staining confirmed that the bundles were actin filaments. Without the addition of the five PSD components but with the presence of cortactin, the Arp2/3 complex, and G-actin, actin bundles were not observed. Finally, it has been shown that PSD can also promote the formation of actin bundles on lipid bilayers in the absence of Arp2/3. Thus, it has been shown that PSD condensates can promote F-actin assembly by increasing the concentration of G-actin and cortactin.

## 5. Conclusions

To date, it is becoming more and more obvious that the LLPS of proteins is of a universal nature and, apparently, mediates all known cellular processes [9]. The process of the formation of F-actin, which is one of the main components of the cytoskeleton, is no exception.

**Figure 4:**
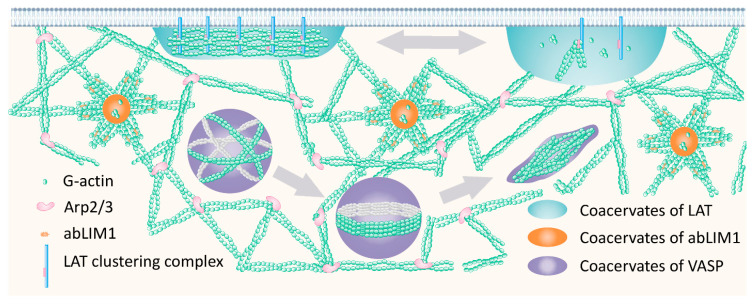
The currently discovered, coacervate-dependent models of actin polymerization and bundling. The figure was created based on the results of the works [2,27,53].

Figure 4 shows the coacervates that are known, to date, to arise from LLPS, which plays a crucial role in actin polymerization. This model shows:

− coacervates of LAT signaling proteins capable of integrating actin-binding proteins, which, as noted in the literature, leads to the intensification of their work (see Section 3);
− coacervates of the actin-binding protein, abLIM1, which integrates G-actin monomers and thereby increases their local concentration, thus leading to the initiation of polymerization and the growth of F-actin fibrils in the form of asters (see Section 2);
− VASP coacervates, which trigger the spontaneous, self-sustaining growth of actin bundles and are analyzed in detail in the yet-unpublished work of Graham et al. [53]. Actin polymerizes inside the VASP droplets, and elongating filaments are distributed along the periphery of the droplet, forming an actin-rich ring inside the droplet. As actin polymerizes and the ring thickens, its rigidity increases and eventually overcomes the surface tension of the drop, deforming the drop and transforming it into a linear bundle. The resulting bundles contain long, parallel actin filaments growing from their tips (Figure 4). The length of the final F-actin bundle can be many times greater than the size of the initial droplet.

Actin filaments are known to be associated with the membrane. Within the framework of new ideas of actin polymerization, it becomes clear that this is energetically favorable since the threshold concentration of coacervate formation on the membrane is an order of magnitude lower than in free space [54]. New ideas about actin polymerization reveal new directions in the study of the cytoskeleton and formulate new challenges for researchers. It remains to be seen whether some other actin-binding proteins can form coacervates during phase separation and under which scenario they will work, similar to how the abLIM1 or VASP coacervates interact with formed filaments [3]. It is interesting to evaluate the existence of proteins that can form coacervates similar to signaling proteins in response to internal cell stimuli.

Together with earlier work on the mechanism of microtubule formation through the concentration of tubulin in centrosomes [4], recent works on actin polymerization highlight the universal role of LLPS in the formation of the cytoskeleton. Researchers are now challenged to explore the dynamic cytoskeletal organization within the frame of this new paradigm.

## Data Availability

Not application.

## References

[1] Li P., Banjade S., Cheng H.C., Kim S., Chen B., Guo L., Llaguno M., Hollingsworth J.V., King D.S., Banani S.F. (2012). Phase transitions in the assembly of multivalent signalling proteins. Nature.

[2] Su X., Ditlev J.A., Hui E., Xing W., Banjade S., Okrut J., King D.S., Taunton J., Rosen M.K., Vale R.D. (2016). Phase separation of signaling molecules promotes t cell receptor signal transduction. Science.

[3] Wiegand T., Hyman A.A. (2020). Drops and fibers—How biomolecular condensates and cytoskeletal filaments influence each other. Emerg. Top. Life Sci..

[4] Woodruff J.B., Ferreira Gomes B., Widlund P.O., Mahamid J., Honigmann A., Hyman A.A. (2017). The centrosome is a selective condensate that nucleates microtubules by concentrating tubulin. Cell.

[5] Shin Y., Brangwynne C.P. (2017). Liquid phase condensation in cell physiology and disease. Science.

[6] Wilson E.B. (1899). The structure of protoplasm. Science.

[7] Brangwynne C.P., Eckmann C.R., Courson D.S., Rybarska A., Hoege C., Gharakhani J., Jülicher F., Hyman A.A. (2009). Germline p granules are liquid droplets that localize by controlled dissolution/condensation. Science.

[8] Uversky V.N. (2017). Intrinsically disordered proteins in overcrowded milieu: Membrane-less organelles, phase separation, and intrinsic disorder. Curr. Opin. Struct. Biol..

[9] Fonin A.V., Antifeeva I.A., Kuznetsova I.M., Turoverov K.K., Zaslavsky B.Y., Kulkarni P., Uversky V.N. (2022). Biological soft matter: Intrinsically disordered proteins in liquid-liquid phase separation and biomolecular condensates. Essays Biochem..

[10] Boeynaems S., Alberti S., Fawzi N.L., Mittag T., Polymenidou M., Rousseau F., Schymkowitz J., Shorter J., Wolozin B., Van Den Bosch L. (2018). Protein phase separation: A new phase in cell biology. Trends Cell Biol..

[11] Hofweber M., Dormann D. (2019). Friend or foe-post-translational modifications as regulators of phase separation and rnp granule dynamics. J. Biol. Chem..

[12] Monahan Z., Ryan V.H., Janke A.M., Burke K.A., Rhoads S.N., Zerze G.H., O’Meally R., Dignon G.L., Conicella A.E., Zheng W. (2017). Phosphorylation of the fus low-complexity domain disrupts phase separation, aggregation, and toxicity. EMBO J..

[13] Snead W.T., Gladfelter A.S. (2019). The control centers of biomolecular phase separation: How membrane surfaces, ptms, and active processes regulate condensation. Mol. Cell.

[14] Banani S.F., Lee H.O., Hyman A.A., Rosen M.K. (2017). Biomolecular condensates: Organizers of cellular biochemistry. Nat. Rev. Mol. Cell Biol..

[15] Alberti S., Gladfelter A., Mittag T. (2019). Considerations and challenges in studying liquid-liquid phase separation and biomolecular condensates. Cell.

[16] Gomes E., Shorter J. (2019). The molecular language of membraneless organelles. J. Biol. Chem..

[17] Dominguez R. (2009). Actin filament nucleation and elongation factors--structure-function relationships. Crit. Rev. Biochem. Mol. Biol..

[18] McCall P.M., Srivastava S., Perry S.L., Kovar D.R., Gardel M.L., Tirrell M.V. (2018). Partitioning and enhanced self-assembly of actin in polypeptide coacervates. Biophys. J..

[19] van der Gucht J., Spruijt E., Lemmers M., Cohen Stuart M.A. (2011). Polyelectrolyte complexes: Bulk phases and colloidal systems. J. Colloid Interface Sci..

[20] Black K.A., Priftis D., Perry S.L., Yip J., Byun W.Y., Tirrell M. (2014). Protein encapsulation via polypeptide complex coacervation. ACS Macro Lett..

[21] Lindhoud S., Claessens M.M. (2016). Accumulation of small protein molecules in a macroscopic complex coacervate. Soft Matter.

[22] Martin N., Li M., Mann S. (2016). Selective uptake and refolding of globular proteins in coacervate microdroplets. Langmuir.

[23] Bubb M.R., Govindasamy L., Yarmola E.G., Vorobiev S.M., Almo S.C., Somasundaram T., Chapman M.S., Agbandje-McKenna M., McKenna R. (2002). Polylysine induces an antiparallel actin dimer that nucleates filament assembly: Crystal structure at 3.5-a resolution. J. Biol. Chem..

[24] Cooper J.A. (1987). Effects of cytochalasin and phalloidin on actin. J. Cell Biol..

[25] Pollard T.D. (1986). Rate constants for the reactions of atp- and adp-actin with the ends of actin filaments. J. Cell Biol..

[26] Nakatani N., Sakuta H., Hayashi M., Tanaka S., Takiguchi K., Tsumoto K., Yoshikawa K. (2018). Specific spatial localization of actin and DNA in a water/water microdroplet: Self-emergence of a cell-like structure. Chembiochem..

[27] Yang S., Liu C., Guo Y., Li G., Li D., Yan X., Zhu X. (2022). Self-construction of actin networks through phase separation-induced ablim1 condensates. Proc. Natl. Acad. Sci. USA.

[28] Li G., Huang S., Yang S., Wang J., Cao J., Czajkowsky D.M., Shao Z., Zhu X. (2018). Ablim1 constructs non-erythroid cortical actin networks to prevent mechanical tension-induced blebbing. Cell Discov..

[29] Roof D.J., Hayes A., Adamian M., Chishti A.H., Li T. (1997). Molecular characterization of ablim, a novel actin-binding and double zinc finger protein. J. Cell Biol..

[30] Chen L., Jiang Z.G., Khan A.A., Chishti A.H., McKnight C.J. (2009). Dematin exhibits a natively unfolded core domain and an independently folded headpiece domain. Protein Sci..

[31] Khanna R., Chang S.H., Andrabi S., Azam M., Kim A., Rivera A., Brugnara C., Low P.S., Liu S.C., Chishti A.H. (2002). Headpiece domain of dematin is required for the stability of the erythrocyte membrane. Proc. Natl. Acad. Sci. USA.

[32] Rana A.P., Ruff P., Maalouf G.J., Speicher D.W., Chishti A.H. (1993). Cloning of human erythroid dematin reveals another member of the villin family. Proc. Natl. Acad. Sci. USA.

[33] Vardar D., Chishti A.H., Frank B.S., Luna E.J., Noegel A.A., Oh S.W., Schleicher M., McKnight C.J. (2002). Villin-type headpiece domains show a wide range of f-actin-binding affinities. Cell Motil. Cytoskelet..

[34] Kadrmas J.L., Beckerle M.C. (2004). The lim domain: From the cytoskeleton to the nucleus. Nat. Rev. Mol. Cell Biol..

[35] Koshino I., Mohandas N., Takakuwa Y. (2012). Identification of a novel role for dematin in regulating red cell membrane function by modulating spectrin-actin interaction. J. Biol. Chem..

[36] Lu Y., Hanada T., Fujiwara Y., Nwankwo J.O., Wieschhaus A.J., Hartwig J., Huang S., Han J., Chishti A.H. (2016). Gene disruption of dematin causes precipitous loss of erythrocyte membrane stability and severe hemolytic anemia. Blood.

[37] Bergeron-Sandoval L.P., Safaee N., Michnick S.W. (2016). Mechanisms and consequences of macromolecular phase separation. Cell.

[38] Jiang H., Wang S., Huang Y., He X., Cui H., Zhu X., Zheng Y. (2015). Phase transition of spindle-associated protein regulate spindle apparatus assembly. Cell.

[39] Wang J., Choi J.M., Holehouse A.S., Lee H.O., Zhang X., Jahnel M., Maharana S., Lemaitre R., Pozniakovsky A., Drechsel D. (2018). A molecular grammar governing the driving forces for phase separation of prion-like rna binding proteins. Cell.

[40] Estes J.E., Selden L.A., Gershman L.C. (1981). Mechanism of action of phalloidin on the polymerization of muscle actin. Biochemistry.

[41] Coué M., Brenner S.L., Spector I., Korn E.D. (1987). Inhibition of actin polymerization by latrunculin a. FEBS Lett..

[42] Campellone K.G., Welch M.D. (2010). A nucleator arms race: Cellular control of actin assembly. Nat. Rev. Mol. Cell Biol..

[43] Chhabra E.S., Higgs H.N. (2007). The many faces of actin: Matching assembly factors with cellular structures. Nat. Cell Biol..

[44] Tobacman L.S., Korn E.D. (1983). The kinetics of actin nucleation and polymerization. J. Biol. Chem..

[45] Zuchero J.B. (2007). In vitro actin assembly assays and purification from acanthamoeba. Methods Mol. Biol..

[46] Derivery E., Gautreau A. (2010). Assaying wave and wash complex constitutive activities toward the arp2/3 complex. Methods Enzymol..

[47] Ho H.Y., Rohatgi R., Lebensohn A.M., Kirschner M.W. (2006). In vitro reconstitution of cdc42-mediated actin assembly using purified components. Methods Enzymol..

[48] Frank D., Frey N. (2011). Cardiac z-disc signaling network. J. Biol. Chem..

[49] Case L.B., Zhang X., Ditlev J.A., Rosen M.K. (2019). Stoichiometry controls activity of phase-separated clusters of actin signaling proteins. Science.

[50] Zeng M., Chen X., Guan D., Xu J., Wu H., Tong P., Zhang M. (2018). Reconstituted postsynaptic density as a molecular platform for understanding synapse formation and plasticity. Cell.

[51] Naisbitt S., Kim E., Tu J.C., Xiao B., Sala C., Valtschanoff J., Weinberg R.J., Worley P.F., Sheng M. (1999). Shank, a novel family of postsynaptic density proteins that binds to the nmda receptor/psd-95/gkap complex and cortactin. Neuron.

[52] Han K., Holder J.L., Schaaf C.P., Lu H., Chen H., Kang H., Tang J., Wu Z., Hao S., Cheung S.W. (2013). Shank3 overexpression causes manic-like behaviour with unique pharmacogenetic properties. Nature.

[53] Graham K., Chandrasekaran A., Wang L., Ladak A., Lafer E.M., Rangamani P., Stachowiak J.C. (2023). Liquid-like VASP condensates drive actin polymerization and dynamic bundling. Nat. Phys..

[54] Ditlev J.A. (2021). Membrane-associated phase separation: Organization and function emerge from a two-dimensional milieu. J. Mol. Cell Biol..

